# Radial Oxygen Loss from Plant Roots—Methods

**DOI:** 10.3390/plants10112322

**Published:** 2021-10-28

**Authors:** Juan de la Cruz Jiménez, Elisa Pellegrini, Ole Pedersen, Mikio Nakazono

**Affiliations:** 1 Laboratory of Plant Genetics and Breeding, Graduate School of Bioagricultural Sciences, Nagoya University, Furo-cho, Chikusa, Nagoya 464-8601, Japan; nakazono@agr.nagoya-u.ac.jp; 2 Department of Agricultural, Food, Environmental and Animal Sciences, University of Udine, Via delle Scienze 206, 33100 Udine, Italy; elisa.pellegrini@uniud.it; 3 The Freshwater Biological Laboratory, Department of Biology, University of Copenhagen, Universitetsparken 4, DK2100 Copenhagen, Denmark; opedersen@bio.ku.dk; 4 UWA School of Agriculture and Environment, Faculty of Science, The University of Western Australia, Perth, WA 6009, Australia

**Keywords:** methylene blue staining, microelectrodes, microsensors, root-sleeving electrodes, planar optodes

## Abstract

In flooded soils, an efficient internal aeration system is essential for root growth and plant survival. Roots of many wetland species form barriers to restrict radial O_2_ loss (ROL) to the rhizosphere. The formation of such barriers greatly enhances longitudinal O_2_ diffusion from basal parts towards the root tip, and the barrier also impedes the entry of phytotoxic compounds produced in flooded soils into the root. Nevertheless, ROL from roots is an important source of O_2_ for rhizosphere oxygenation and the oxidation of toxic compounds. In this paper, we review the methodological aspects for the most widely used techniques for the qualitative visualization and quantitative determination of ROL from roots. Detailed methodological approaches, practical set-ups and examples of ROL from roots with or without barriers to ROL are included. This paper provides practical knowledge relevant to several disciplines, including plant–soil interactions, biogeochemistry and eco-physiological aspects of roots and soil biota.

## 1. Introduction

During flooding, the gas-filled pore spaces in the soil that normally facilitate O_2_ diffusion are filled with water and the diffusion of gases (e.g., O_2_) is highly impeded (diffusion coefficient of O_2_ in water is 10,000 lower than in air; [1]). The O_2_ dissolved in soil water or any entrapped air is rapidly consumed by aerobic microorganisms and roots, leading to soil anoxia or severe hypoxia [2,3]. Since O_2_ is unavailable for radial entry into the root system in flooded conditions, longitudinal O_2_ transport between shoot and root takes place and is essential for root respiration and plant growth [1,4]. The effectiveness of internal root aeration via longitudinal O_2_ diffusion depends upon the amount of pore space resistance, the O_2_ consumption by tissues along the diffusion path, the path length (root length) and the formation of tissue barriers to restrict ROL to the rhizosphere [5]. The formation of barriers to ROL in roots is paramount given its dual benefit of preventing ROL to the reduced rhizosphere (high soil O_2_ demand can result in high O_2_ loss from roots, [6]) and the entry into the root of phytotoxic concentrations of substances produced in flooded soils (e.g., Fe^2+^, [7]).

**Figure 1:**
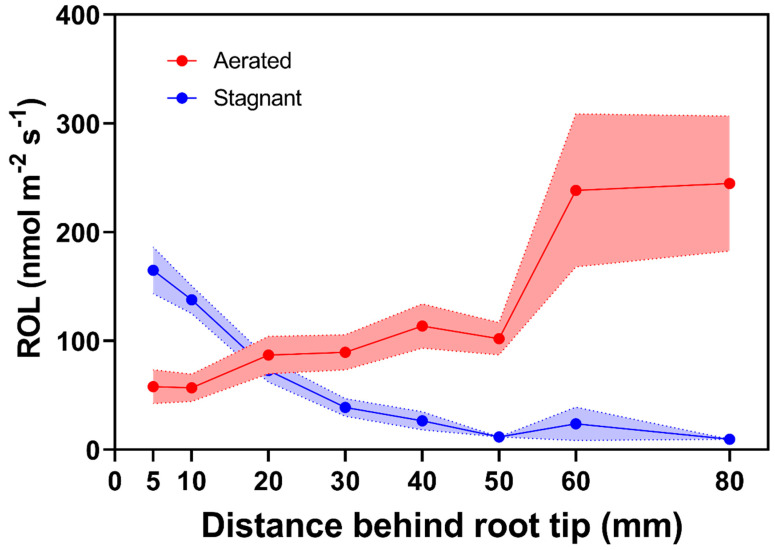
Radial O_2_ loss from individual adventitious roots of rice grown in aerated or stagnant, deoxygenated conditions. In aerated nutrient solution, rice does not form an ROL barrier (with the exception of some wild accessions [20]), whereas the barrier is formed in stagnant, deoxygenated nutrient solution. Points indicate mean radial O_2_ loss at different distances behind the root tip and bands represent standard error (figure was constructed compiling published information on ROL from rice roots [11,12,13,14,15,16,17,18,19,20], *n* = 28). Plants were grown in aerated or stagnant, deoxygenated conditions for 5 to 31 days. Lengths of the roots were 93 to 159 mm.

The presence of barriers to restrict ROL in roots was initially visualized in experiments indicating interspecific differences in root O_2_ flux and marked basipetal reductions in root O_2_ permeability in a number of wetland species [8,9]. Moreover, lower O_2_ radial flux rates were obtained in roots of plants grown in flooded conditions in comparison to aerated controls [10]. For rice roots grown in aerated conditions, higher basipetal ROL rates decreasing towards the root tip are characteristic patterns indicating roots with ‘weak’ or absent barriers to ROL. In contrast, for rice roots that can form barriers to ROL when grown in stagnant, deoxygenated conditions, lower ROL rates along basal zones and higher ROL rates toward the root tip indicate the presence of a tight barrier to ROL [11,12,13,14,15,16,17,18,19,20] Figure 1. The presence of tight barriers to ROL allows for O_2_ diffusion towards the root tip where molecular O_2_ enables root growth and nutrient uptake in these flooded conditions [21,22].

In wetland plants, ROL from roots to the flooded soil occurs especially around the highly O_2_-permeable root tips without barriers [4,23,24]. Moreover, considerable O_2_ loss can originate from developing young roots and basal laterals [25,26], although the lateral roots of rice have recently been shown to also form a barrier to ROL when plants are grown in stagnant, deoxygenated solutions [27]. Given that newly formed and short roots lack barriers to ROL [13,18,28], the sediment oxygenation increases proportionally as the numbers of roots produced by plants in anaerobic soils increase [6]. Sediment oxygenation is important for the oxidation of chemically reduced compounds such as Fe^2+^, Mn^2+^, H_2_S, and CH_4_ [1,29,30]. Moreover, O_2_ release from roots into the flooded soils can influence the redoximorphic biogeochemistry by establishing a spatiotemporal microoxic zone of potentially high microbial activity [31,32]. The amount of O_2_ taken up by plant shoots and released from roots to flooded soils depends upon many above and belowground factors, including the numbers, types (e.g., adventitious, laterals) and lengths of roots, the magnitude and distribution of their pore-space and tissue respiratory demand, the degree and distribution of barriers to impede ROL, the numbers of the aerial shoots in capacity for O_2_ uptake, the porosity and O_2_ demand within these shoots, their lengths and the degree of submergence of the aerial shoots, the submerged soil O_2_ demand, their microbial activity, their physical properties (i.e., O_2_ diffusivity being lower in clay than sandy soils) and temperature [2,6].

Within the past few decades, active research has been conducted on the ecological importance of ROL to the flooded soil as well as the anatomical location, chemical characterization, interspecific differences and external triggers for barriers to impede ROL from roots [7,11,12,15,16,17,33,34,35,36,37,38,39]. In this review, we summarize the main methodological techniques used for the visualization and quantification of root ROL. The different techniques described in here have been instrumental for our current knowledge of O_2_ dynamics in submerged substrates. Understanding the strengths and weaknesses of the different methods for ROL quantification as well as the potential uses of each method will provide the needed information for an appropriate method selection and accurate quantification of ROL from roots of plants grown in diverse conditions.

## 2. Methods for Root Radial O_2_ Loss Determination

Radial O_2_ loss from roots can be qualitatively visualized and/or quantitatively determined using several contrasting approaches (Table 1). Here, we describe the most widely used methods for ROL measurements reported in literature. We focus on the principles, main uses and limitations as well as practical set-ups for each method. Detailed protocols and materials needed for each method are provided as Supplementary Information (Supplementary S1–S4). Other methods and approaches for determination of related aspects to ROL including sediment redox potential, iron plaques on roots and mathematical models are summarized in [4].

### 2.1. Qualitative Colorimetric Methods

The colorimetric methods for ROL determination are based on the principle of the oxidation-reduction of an external molecule that reacts with molecular O_2_. Changes in coloration can be visualized and be indicative of ROL from roots. Colorimetric methods for ROL determination include the indigo-carmine [40], methylene blue [25,37], Ti^3+^-citrate [41,42], the anthraquinone radical anion [43] and crystal violet [44].

#### 2.1.1. Principle of the Method

**Figure 2:**
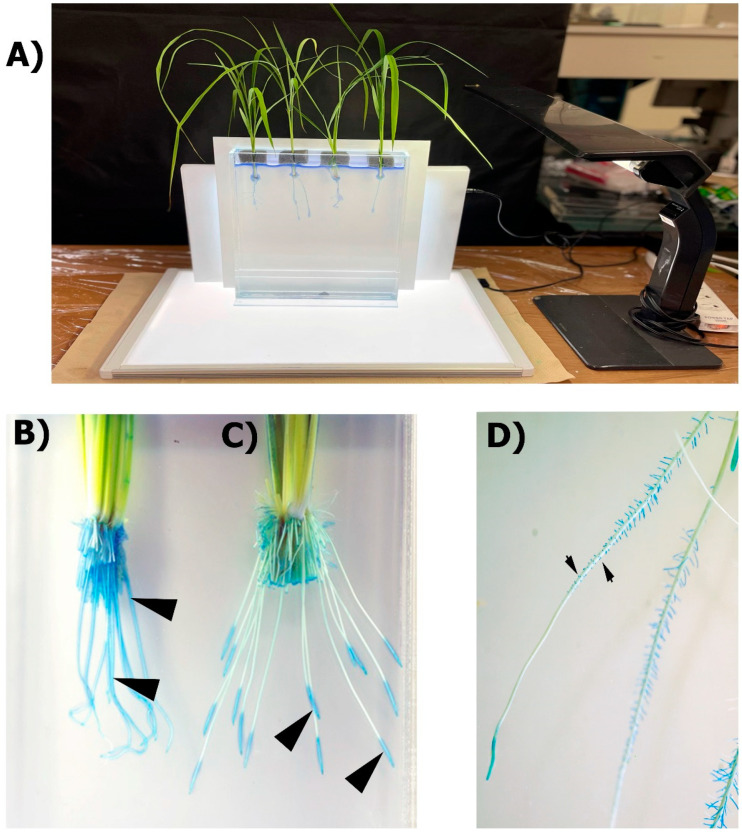
Colorimetric methylene blue method for root radial O_2_ loss determination. (**A**) General set-up including photo chamber filled with deoxygenated solution and reduced methylene blue dye. Intact rice plants are inserted and fixed to the photo chamber. Light panels in A are provided to ensure photosynthesis and internal O_2_ transport and for enhanced photo collection. (**B**) Rice roots with no barriers to ROL. (**C**) Rice roots with barriers to ROL. (**D**) Sites of O_2_ loss through root ‘windows’ of developing laterals (arrows). Rice plants in A and B were grown for 28 days in aerated or stagnant, deoxygenated solutions, respectively. Rice plants in D were grown for one week in aerated solutions with high concentration of Fe (300 µM). Arrowheads in B and C point to blue halos where O_2_ loss is occurring.

The methylene blue is the most widely used method for the colorimetric determination of ROL from roots. The methylene blue dye is bright blue in the oxidized state and colorless in the reduced state. This dye has been used to study the sites of ROL from roots of plats grown in different conditions including stagnant, deoxygenated solutions [25,37] and flooded soils [33]. Once the methylene blue dye is reduced in a deoxygenated medium, the sites of root ROL can be detected as an intense blue color along these regions of the roots where O_2_ loss occurs (Figure 2).

#### 2.1.2. Practical Set-Up

Root systems of intact plants are completely submerged in a container filled with deoxygenated agar solution and the “colourless” reduced methylene blue (Figure 2A). The root–shoot junction is submerged a few centimeters (i.e., 2 cm) down into the reduced solution to avoid O_2_ leaks in that zone while the shoots remain in air. The plant is fixed and ROL is visualized after c. 30 min by blue coloration in the rhizosphere or on the root surfaces (see protocol in Supplementary Information S1). Roots with a weak barrier, or no barrier at all, will display blue coloration longitudinally across the entire root (Figure 2B). In contrast, roots with a strong barrier to ROL only show blue halos at the root tip where there is no barrier formation (Figure 2C). The resolution of the technique is sufficient to indicate the sites of ROL produced by root ‘windows’ of developing laterals (Figure 2D).

#### 2.1.3. Generalities

The methylene blue method is appropriate for a rapid and initial screening of the main sites of root ROL or the visualization of a barrier to ROL in roots. This inexpensive method has been used to determine differences in ROL among genotypes from a large population (e.g., recombined inbred lines of maize, [37]) or for a rapid determination of ROL on different roots from the same genotype (e.g., rice, [18,44]). The main limitations of the methylene blue method are (1) its qualitative nature and (2) the unknown O_2_ detection limit (Table 1). This will restrict the method use to a simple characterization of the sites of ROL and/or the presence/absence of barriers to ROL instead of detailed physiological studies on root ROL barrier strength.

### 2.2. Polarographic Cylindrical Oxygen Electrode

#### 2.2.1. Principle of the Method

**Figure 3:**
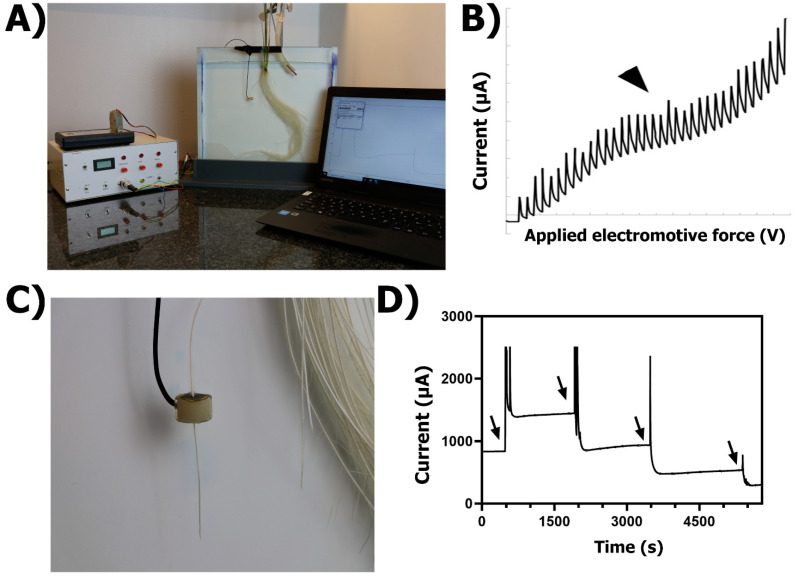
Polarographic method for radial O_2_ loss determination in roots. (**A**) General set-up including a transparent acrylic chamber filled with deoxygenated solution and the roots of an intact rice plant submerged. Different electrodes (see text for details) are inserted into the deoxygenated solution and connected to a polarograph. (**B**) Current–voltage curve for O_2_ determination in solutions. (**C**) Close-up of a root inserted into a Pt-sleeving electrode. (**D**) Diffusion current from the Pt-sleeving electrode. Arrowhead in B points to current plateau where the rate of O_2_ reduction is independent of voltage and only depends on the rate of O_2_ diffusion to the cylindrical electrode surface. Arrows in D indicate the points when Pt-sleeving electrode was moved in 10 mm steps from basal parts toward the tip of a root of *Hordeum marinum* grown in aerated solutions. Note the time taken for current stabilization before a new measurement.

The polarography is an analytical technique based on the electro-oxidation or electro-reduction of substances in solution under an applied electromotive force. This technique utilizes the characteristics of the current–voltage curve for O_2_ identification and the current output to quantify its amounts. The polarographic O_2_ determination in a deoxygenated medium requires an electrical circuit where a cathode, a cylindrical platinum electrode (syn. sleeving-electrode), is connected to an anode (e.g., reference electrode; usually Calomel or Ag/AgCl electrode) and these two to an amperometer (Figure 3A). The sleeving-electrode is polarized in the deoxygenated solution before measuring ROL from a root (see protocol in Supplementary Information S2). Polarization voltage is applied to the circuit and a current plateau placed somewhere between −0.2 and −0.8 V indicates that the rate of O_2_ reduction is independent of voltage and only depends on the rate of O_2_ diffusion to the cylindrical electrode surface ([9]; Figure 3B). The current plateau depends upon the amount of O_2_ present in the solution. Under the appropriate polarizing voltage, the sleeving-electrode acts as a sink for O_2_, (the O_2_ concentration at the electrode surface is effectively maintained at zero) and a diffusion gradient is maintained between root and electrode [1,45].

#### 2.2.2. Practical Set-Up

The root-sleeving electrode and the root system of an intact plant are immersed in an acrylic chamber filled with stagnant, deoxygenated solution (Figure 3A). Once the sleeving-electrode is polarized, a root of an intact plant is inserted through the cylindrical Pt electrode (Figure 3C). The electrode can be positioned at different distances along the root and the diffusion current at a given position on the root is measured after the current has equilibrated (c. after 25 min, Figure 3D). After measuring the root, the residual current in the deoxygenated solution is obtained by placing the electrode away from the root and measuring the current at equilibrium. The rate of ROL from the root at equilibrium can be calculated using the equation proposed by Armstrong and Wright [45]:

(1) $$\mathrm{ROL}=\frac{4.974\times I\times 10^{4}}{\left(A\times 60\times 32\right)}$$

where ROL is the radial O_2_ loss (O_2_, nmol m^−2^ root surface s^−1^), I is the diffusion current (µA) with the root in the electrode minus the residual current (µA) in the deoxygenated solution without a root and A is the surface area of the part of the root within the electrode (cm^2^). The aboveground parts of the plants remain above water and photosynthetically active radiation is provided to ensure stomatal opening and O_2_ transport from shoot to roots. The ROL is usually measured at different positions along the root. ROL decreasing towards the root–shoot junction (the source of the O_2_) indicates the presence of a barrier to ROL (Figure 1, cf. [12,24]). In contrast, higher basipetal ROL rates decreasing towards the root tip are characteristic patterns of roots with ‘weak’ or absent barriers to ROL (Figure 1).

#### 2.2.3. Generalities

The polarographic determination of ROL is appropriate to accurately detect very small amounts of O_2_ leaking from roots. Moreover, this technique can be used to detect values of high ROL rates (i.e., c. 21 kPa pO_2_). However, incomplete reduction of O_2_ at the electrode surface can occur when artificially perfusing root sections with high pO_2_ (i.e., pO_2_ > 38.7 kPa, [46]). Therefore, care must be taken when evaluating ROL at different O_2_ concentrations surrounding the shoot. The main limitations for this method include the use of custom-built equipment and the long time required to obtain ROL rates (Table 1).

### 2.3. Clark-Type O_2_ Microelectrodes and O_2_ Micro-Optodes

#### 2.3.1. Principle of the Method: O_2_ Microelectrode

**Figure 4:**
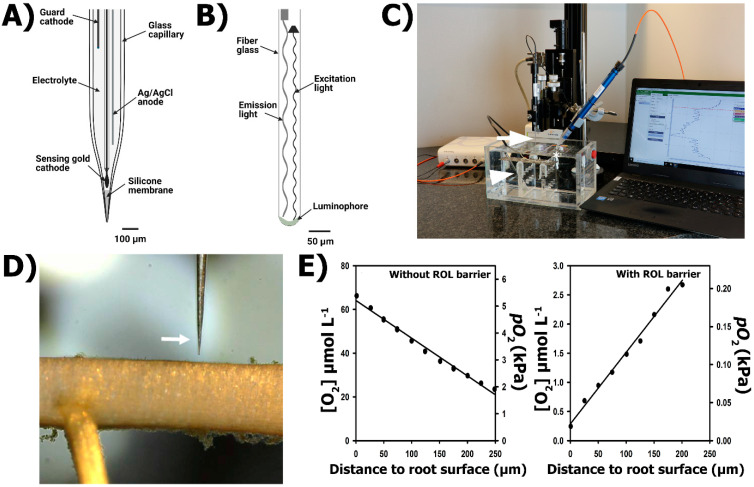
O_2_ microelectrodes and O_2_ micro-optodes for ROL determination from roots. (**A**) Clark-type O_2_ microelectrode with guard cathode. (**B**) O_2_ micro-optode. (**C**) General set-up including a transparent acrylic chamber filled with deoxygenated solution and the submerged roots (detached) of a rice plant. The O_2_ microelectrode (to obtain spatial concentration profiles) and the O_2_ micro-optode (to follow the O_2_ in the bulk) are mounted in a motorized stage and connected to a picoammeter and an optode meter, respectively. (**D**) Close-up of an O_2_ microelectrode near the root surface of *Zea nicaraguensis*. (**E**) Characteristic O_2_ gradients from roots without a barrier (roots from *Zea nicaraguensis* plants grown in aerated nutrient solutions for 25 days, left) and a tight barrier to ROL (roots from *Zea nicaraguensis* plants grown in staganant, deoxygenated nutrient solutions for 25 days, right). Arrow in C points to O_2_ microelectrode, arrowhead to temperature sensor and asterisk key to O_2_ micro-optode. Arrow in D shows the tip of an O_2_ microelectrode.

The O_2_ microelectrodes are amperometric sensors where an anode and a cathode are fused into one sensor [47], resembling a miniaturized version of the Clark-type sensors [48]. The O_2_ quantification at the microelectrode tip relies upon O_2_ diffusion from the medium to an O_2_-reducing cathode, which is polarized against an internal anode. An O_2_ microelectrode contains a gold-coated sensing cathode, a silver or gold wire guard cathode (reducing cathode) and an Ag/AgCl anode, all inserted into a glass capillary filled with an electrolyte solution and sealed at the tip with a gas-permeable silicone membrane ([47]; Figure 4A). The electrodes inside the glass capillary form a circuit where the resulting signal is in the pA (10–12 A) range. A picoammeter is used to polarize the circuit and to amplify the current output. The guard cathode removes O_2_ in the electrolyte, minimizing zero current, response time and polarization time. When the zero current has stabilized following a period of polarization, the current in the measuring circuit depends only on the diffusional supply of O_2_ from the medium to the surface of the microelectrode membrane [47]. The response to O_2_ is linear all the way up to pure O_2_, i.e., 101 kPa pO_2_.

#### 2.3.2. Principle of the Method: O_2_ Micro-Optodes

The O_2_ micro-optodes are basically a fiber glass coated with an oxygen-quenchable luminophore (Figure 4B). The luminophore is excited when illuminated at a particular wavelength. Oxygen can act as a dynamic luminescence quencher, decreasing the luminescence quantum yield of the luminophore and therefore the O_2_ concentration can be determined by the difference resulting from the amount of emitted light by the luminophore in presence and absence of O_2_ [49,50]. The response to O_2_ is non-linear but modern meters handle the non-linear response based on a 2-point calibration, typically at zero O_2_ and with O_2_ at air equilibrium. Oxygen optodes lose resolution with increasing O_2_ partial pressure and most sensors should not be used for pO_2_ exceeding 40 kPa.

#### 2.3.3. Practical Set-Up

Radial O_2_ loss from roots to the external medium can be quantified by inserting roots of intact plants (or detached root sections) into stagnant, deoxygenated solution. The O_2_ flux is measured at the root surface as well as a few µm (typically 200 µm) away from the root surface (deoxygenated medium) by either O_2_ microelectrodes or micro-optodes (see protocol in Supplementary Information S4). For this purpose, the microsensor (O_2_ microelectrode or micro-optode) needs to be fixed to a micro-manipulator fitted on a motorized stage to control micrometrical movement from the deoxygenated solution towards de root (Figure 4C). The root is mounted horizontally in a fine woven wire mesh and held by plastic bands attached to the mesh and mesh with the root mounted is submerged into the deoxygenated solution. The microsensor movement is controlled remotely and visualized by stereo microscope to guarantee that the microsensor tip reaches the root surface at the target point for ROL determination (Figure 4D). Stirring near the root must be uniform in order to reproduce accurate determinations of O_2_ fluxes from roots to medium (ROL) or from medium to roots (O_2_ consumption by roots). Radial O_2_ loss from roots can be calculated from the slope of the O_2_ gradient from the root surface into the deoxygenated medium using the equation proposed by Henriksen [51]:

(2) $$\mathrm{ROL}=2\pi D\frac{\left(C2-C1\right)}{\ln\left(\frac{r2}{r1}\right)}$$

where *D* is the O_2_ diffusion coefficient in the agar medium (approximates that in water at the experimental temperature), *C*1 is the O_2_ concentration at the root surface (mol m^−3^) calculated from the partial pressure, *C*2 is the O_2_ concentration at a given radial distance away from the root surface *r*2 (m) and *r*1 is the radius of the root (m). ROL measurements can be conducted along the root when the aboveground parts of the plant are above water (i.e., as explained before for ROL measurements using root-sleeving electrodes) or can be conducted on excised root segments sealed with lanoline in both cut ends [52]. Provided that the main source for O_2_ is O_2_ from root aerenchyma (deoxygenated solutions still have traceable amounts of O_2_), the slope of the O_2_ gradient from the deoxygenated medium into the root surface determine the presence of barriers to ROL. Oxygen gradients increasing towards the root surface indicate O_2_ release and thus that no barrier to ROL is present, or if present, the barrier is weak (Figure 4E). In contrast, a strong barrier to ROL is present when dissolved O_2_ decreases towards the root surface (Figure 4E) as no O_2_ is being released by roots. The patterns of ROL obtained with O_2_ microsensors might be similar to those obtained using the root-sleeving electrode (e.g., Figure 1; [28]), but in many cases the measured fluxes are lower when obtained by a microsensor. The root-sleeving electrodes reduce all O_2_ at the Pt surface and therefore the O_2_ gradient is steeper inside the ring, resulting in a higher flux.

#### 2.3.4. Generalities

Oxygen microelectrodes and O_2_ micro-optodes are very sensitive to low O_2_ concentrations (nanomolar range), making them appropriate tools for ROL determinations. Moreover, the narrow tip diameters (3–10 μm for Clark-type microelectrodes and 50 μm for micro-optodes) allow for the spatial resolution of O_2_ gradients to tissue and cell levels. The microelectrodes have been instrumental to obtaining O_2_ profiles inside plant tissues, including the visualization of anoxic cores inside roots [53,54]. The higher spatial resolution of the O_2_ microelectrode and O_2_ micro-optodes, however, can also be a weakness for root ROL characterization as permeable cells (windows) and emerging laterals can cause O_2_ leaks to the rhizosphere (Figure 2D; [7,20,53,55]).

The O_2_ microelectrodes with guard cathodes have almost undetectable zero currents of <1 pA and 90% response times in less than 200 ms [47,56]. O_2_ microelectrodes have a faster response and smaller tip diameter than O_2_ micro-optodes, but O_2_ microelectrodes require calibration more often, especially when changes in temperature occur (Table 1). The development of micro-optodes with a smaller diameter than 40 μm is limited due to a dramatic decrease in luminescence signal reducing O_2_ sensitivity [49]. Due to its working principle, an O_2_ microelectrode consumes an extremely low amount of O_2_ (ca. 4 × 10^−4^ nmol O_2_ h^−1^, [57]), causing a ca. 1–2% signal difference between the signal in stagnant media compared to turbulent media or media with high diffusivity such as gasses. In contrast, the O_2_ micro-optode is insensitive to stirring of the media due to its non-oxygen consuming detecting principle. The stiffness of the glass capillary makes the microelectrode suitable to penetrate plant tissues, whereas the flexible nature of the optical fiber of the O_2_ micro-optode makes it unsuitable for such measurements [50]. The signal of both microsensors can be affected by some other chemical compounds and strong organic solvents (Table 1).

### 2.4. Planar Optodes

#### 2.4.1. Principle of the Method

The planar optode is a relatively recent tool for the 2D visualization and quantification of O_2_. The planar optode uses the same principle of an O_2_ quenchable luminophore, as explained above for the O_2_ micro-optode, but the signal intensity (O_2_-dependent) is photographed and analyzed, allowing for both the visualization and quantification of O_2_ dynamics in 2D.

#### 2.4.2. Practical Set-Up

**Figure 5:**
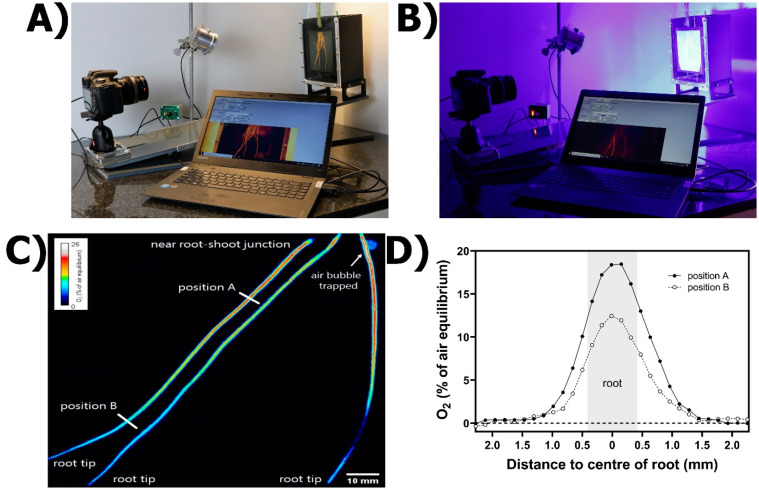
Planar optodes for ROL determination from roots. (**A**) General set-up, including a chamber filled with anoxic medium and the roots of an intact rice plant submerged, a digital camera, blue LED lights and software for image analysis (see text and Supplementary Information S4 for details). (**B**) Image collection process in darkness. (**C**) O_2_ planar optode image indicating differences in ROL in roots of *Puccinellia festuciformis* grown in aerated conditions for 5 weeks. (**D**) ROL quantification in the regions of interest. Oxygen concentrations in (**D**) refer to specific positions of the root, as shown in (**C**).

The practical set-up for the planar optode method consists of a commercial regular RBG camera with an emission filter (530 nm) attached, an O_2_-sensitive luminophore (indicator dye), a light-harvesting dye (antenna), and a high-power blue LED light with an excitation filter (435 nm; [58] Figure 5A). The indicator and antenna dyes are dissolved in a permeable matrix and coated into a dust-free, transparent polyester foil to form the O_2_-sensing planar optode (see details on methodological aspects in Supplementary Information S4). A black silicone layer is added to reduce light scattering and reflection from any background behind the planar optode [58]. In water-saturated systems, the planar optode is placed near the surface of the soil/sediment or next to roots inside a transparent-walled aquarium. The RGB camera is positioned perpendicular to the planar optode and LED lights are orientated at a 45° angle to the camera (Figure 5A; [58,59]).

Oxygen distribution within the planar optode is evaluated by illuminating the planar optode with the specific LED light, and the luminescence emitted is recorded in a photograph. Measurements are conducted in dark conditions to avoid light interference (Figure 5B). The main approaches for image analysis include luminescence intensity, ratiometric imaging and luminescence lifetime. Luminescence intensity measurements have some disadvantages as they are sensitive to variations in background reflection, inhomogeneous distribution of the luminophore and variations in the homogeneity of the excitation light [60]. Luminescence lifetime imaging offers some advantages, as this technique allows for excellent contrast enhancement and background suppression of unwanted luminescence in the image [61,62]. However, luminophores with relatively long lifetimes and complex hardware and software for triggering and synchronizing image acquisition and sensor excitation are required [60]. The ratiometric imaging approach is therefore a preferred technique, as this method is simple, inexpensive and reduces the noise of the image using differences in luminescence intensity between the antenna dye (does not react with O_2_; therefore, luminescence intensity is unaffected) and the indicator dye (emission quenched in the presence of O_2_) as an internal reference for the O_2_ sensor [58]. Color patterns in the planar optode image indicate differences in O_2_ concentrations at the interface between roots and anoxic sediment, highlighting longitudinal gradients of ROL (Figure 5C). Radial O_2_ loss can be quantified in selected regions of interest in the picture, based on O_2_ extinction/depletion between root surface and the anoxic sediment using open-source software (Figure 5D; see details in Supplementary Information S4).

#### 2.4.3. Generalities

The majority of studies using planar optodes have a focus on benthic interactions in marine sediments [30,58,59,63,64]. This technique is also used for evaluating spatio-temporal variability in O_2_ dynamics in waterlogged crops (e.g., rice; [65]). Spatial resolution in planar optodes is largely determined by image format, pixel size, optical lenses and the camera’s field of view [60]; using an appropriate set-up, maximum spatial resolutions can be comparable to that obtained with the smaller O_2_ microelctrode (e.g., <10 µm^2^). Nonetheless, planar optodes can resolve O_2_ dynamics in larger areas of the root and soil interfaces as compared to O_2_ microsensors [66,67]. Response time of the planar optodes depends upon the thickness of the layers and the silicone coating [59]. Moreover, the optode response is nonlinear, with maximum sensitivity at low O_2_ concentrations for most of the indicators, but still sufficient accuracy from 50% to 100% of air equilibrium (up to 20.6 kPa pO_2_) [58]. The main constraint for using planar optodes is that O_2_ quantification and imaging are being conducted throughout and along a glass wall that interferes with the root radial O_2_ diffusion. Placing an O_2_-impermeable barrier along a root will enlarge the oxygenated area as compared to a situation without such a wall cf. [68]. Moreover, light scattering properties can be affected by several factors including the wall material, the planar optode itself, background light, a thin water film or air bubbles trapped between optode and wall and the silicone coating; potentially generating a skewed and smeared image of the O_2_ distribution around the root [68,69]. These issues can be minimized or even eliminated by an appropriate selection of materials and calibration procedures [69]. A comprehensive list of existing O_2_ indicators, including critical photophysical and sensing properties and the criteria for indicator selection, is summarized in Quaranta et al. [70]. Another important aspect for planar optode use is that good contact between the sensor foil and the root is needed to ensure optimal luminescence and O_2_ quantification. Imaging in focal planes is ideal, as the further away from the actual roots the picture is taken, the lower the obtained O_2_ concentration [71], but obtaining full contact of roots with the optode wall is often challenging as the root architecture and geometry varies. However, recent developments in optical O_2_ sensor nanoparticles have allowed for a full visualization and quantification of O_2_ at different focal planes of the whole rizhosphere level when using artificial transparent sediment [71].

## 3. Conclusions and Further Perspectives

Much remains to be learnt regarding radial O_2_ loss from roots and the associated redox processes occurring in submerged substrates. The contribution of radial O_2_ loss to important bio-geochemical processes, including methane production/consumption, the oxidation of metals, the solubilization of metals, pH dynamics, and the development of microoxic zones for microbial activity, as well as possible drawbacks of the presence of barriers to radial O_2_ loss in agricultural crops, remains unclear. Different methods for qualitative and/or quantitative determinations of ROL from roots have been fundamental to our current understanding of this important biological process. Technical and practical knowledge, as well as the production of custom-made equipment, has been the main limitation for a broader use of these techniques for ROL determination in ecological studies. However, recent research progress, method development and the commercially available equipment needed for ROL determinations have opened the possibility to increase our current understanding of this topic. The integration of different techniques and analyses appears to be an excellent option to unravel complex chemical processes in submerged substrates. For example, the combined use of O_2_ planar optodes with diffusive gradients in thin films has been successfully used to demonstrate higher metal mobilization in a geochemical niche of higher O_2_ concentration and low pH adjacent to root tips of rice [72]. Moreover, the recent development of hyperspectral luminescence imaging, together with signal deconvolution analysis, enabled the development of a multi-indicator, high-resolution approach with simultaneous imaging of multiple analytes [73]. The techniques described here have been proved to be instrumental for a wide range of uses, from large population screening studies to single cell O_2_ determination. The combined use of the techniques described here would be key for understanding internal O_2_ dynamics and oxidation processes in wetland ecosystems. Moreover, the integration of the techniques for ROL determination with other cutting-edge technologies for plant phenotyping would be essential to study how plants develop and respond to adverse environmental conditions.

## Figures and Tables

**Table 1:** Qualitative and quantitative methods for determination of radial O_2_ loss from roots.

Technique	Indication	Advantages	Disadvantages
Methylene blue staining	Qualitative	Cheapest and quickest method; rapid screening approach for a large number of plants	Qualitative nature; unknown detection limit
Polarographic cylindrical O_2_ electrodes	Quantitative	Accurate quantification of very small amounts of radial fluxes of O_2_	Use of custom-built equipment is necessary; frequent calibration is needed; time-consuming method
Clark-type O_2_ microelectrodes	Quantitative	Commercially available; rapid and accurate O_2_ quantification; linear response to O_2_; resolution to tissue and cell level; appropriate for penetration into tissues	Relatively short lifetime (especially in <10 µm sensors); expensive equipment and frequent calibration needed; signal can be affected by chemical compounds and electrical noise
O_2_ micro-optodes	Quantitative	Commercially available; rapid and precise O_2_ quantification; resolution to tissue level; long lifetime	Possible interferences with inorganic substances; low signal to noise ratio at high O_2_ concentrations; not suitable for penetration into tissue
Planar optodes	Qualitative and Quantitative	Quantitative and qualitative 2D determination of O_2_	Custom-built equipment and technical knowledge needed; non-linear response to O_2_

## Data Availability

Not applicable.

## Supplementary Materials

The following are available online at https://www.mdpi.com/article/10.3390/plants10112322/s1: Supplementary information S1: methylene blue protocol, Supplementary information S2: root-sleeving electrodes protocol, Supplementary information S3: O_2_ microsensors protocol, Supplementary information S4: planar optodes protocol.
