# Supplementary material for: Radial Oxygen Loss from Plant Roots—Methods
Source: Plants (Basel). 2021 Oct 28;10(11):2322. doi: 10.3390/plants10112322 (PMC8622749; doi:10.3390/plants10112322)
Supplement: Supplementary file 1 [file plants-10-02322-s001.zip › plants-1437146-supplementary.pdf]

Jiménez JdlC, Pellegrini E, Pedersen O, Nakazono M. ***Radial oxygen loss from plant roots – methods - Supplementary information***

Supplementary information S1: Methylene blue method

List of equipment needed

- Conical flask
- Transparent acrylic photo chamber
- Digital camera
- PAR lights
- Light panels
- Autoclave

List of chemicals needed

- High-purity N_2 gas
- Agar
- Methylene blue (indicator)
- Sodium dithionite (reducing agent)

General procedure

- *Prepare 0.1% (w/v, 1 g/L) agar in deionized water* by autoclaving at 120 °C for 30 min. Magnetic stirring until the temperature of the solution has fallen below 60 °C prevents formation of clumps.
- *Once the solution is at room temperature, purge this with N_2 for ca. 30 min to remove as much O_2 as possible*. Use a cylinder or conical flask and seal it with wet paper towel during purging with N_2 to avoid air mixing.
- *Add methylene blue to a final concentration of 0.03 mM*.
- *Immediately add sodium dithionite to a final concentration of 0.3 mM*.
- *Mix well but gently.* The solution is initially bluish but will turn clear once sodium dithionite has reduced the methylene blue in the solution. Sometimes blue spheres remain visible after mixing; these will disappear after some times when bubbling the solution with N_2. Alternatively, these can be removed using an eyedropper pipette.
- *Siphon the clear solution into the photo chamber*. Make sure not to form bubbles when siphoning. Steps 3 through 5 can be directly done at the photo chamber.
- *Select a suitable plant and identify the target root(s).* Cut off all other roots near the root-shoot junction to avoid that these roots oxygenize the medium
- *Position a plant*. The shoot base should be 1-2 cm inside the solution. The shoot can be fixed with foam. Make sure that the plant does not move during analysis.

- *Once the plant is fixed, inspect the staining and take a photo*. Wait for 20 min and take photo of the roots. Wait for another 20 min and take new photo. The procedure takes around 1 h, but this can vary according to radial O_2 loss rates from different plant species.

General considerations

- Illumination is key. Make sure to use an appropriate set-up with diffuse light to avoid shadows. The use of light panels both below and behind the roots give good contrast for photo collection.
- Provide PAR illumination and leave the shoot of the plant intact to guarantee photosynthesis and O_2 transport.
- Adding extra reducing agent into the solution will delay the appearance of blue halos surrounding the root.
- The use of macro lens is recommended to get high quality close-up photos.
- The reducing agent (sodium dithionite) is toxic!

Relevant literature

- Armstrong J, Armstrong W. 1988. *Phragmites australis* – A preliminary study of soil-oxidizing sites and internal gas transport pathways. *New Phytologist* 108: 373–382.

- Yamauchi Y, Abe F, Tsutsumi N, Nakazono M. 2019. Root cortex provides a venue for gas-space formation and is essential for plant adaptation to waterlogging. *Frontiers in Plant Sciences* 10: 259. doi: 10.3389/fpls.2019.00259

Supplementary information S2: Sleeving electrodes method

List of equipment needed

- Cylindrical (sleeving) platinum electrode
- Reference electrode (e.g., calomel or Ag/AgCl electrode)
- Transparent acrylic chamber with a lid
- Polarogram
- Chart recorder
- PAR lights
- Digital calliper

List of chemicals needed

- High-purity N_2 gas
- Agar
- Potassium chloride (KCl)
- Calcium sulphate ($CaSO_4$)

General procedure

- *Prepare 0.1% (w/v, 1 g/L) agar in deionized water* by autoclaving at 120 °C for 30 min. Magnetic stirring until the temperature of the solution has fallen below 60 °C prevents formation of clumps.
- *Once the solution is below 40 °C, add KCl to a final concentration of 5mM and also add $CaSO_4$ to a final concentration of 0.5 mM.* KCl ensures adequate electrical conductivity and $CaSO_4$ facilitates root growth.
- *Purge this solution with high-purity N_2 for c. 30 min to remove O_2*. Use a cylinder or conical flask and seal it with wet paper towel during purging with N_2 to avoid mixing atmospheric O_2 back into the solution.
- *Syphon the solution into the transparent acrylic chamber to the top surface.* Care must be taken to ensure O_2 is not introduced back into the solution.
- *Fit a lid to the chamber and seal the holes with moist cotton wool to slow down the entry of air.*
- *Dip the cylindrical electrode into the medium*. Air bubbles often get trapped within the electrode, so agitate gently up and down to help remove any bubbles. Care should be taken not to mix air into the solution.
- *Carefully insert the plant roots into the anoxic medium*. The shoot base should be ca. 1 cm inside the solution. The shoot can be fixed with foam or wet cotton wool.
- *Insert the reference electrode*.
- *After reaching equilibrium (ca. after 30 min) run a polarogram with the sleeving electrode free in the stagnant solution* (do not insert the root at this stage).

- *Identify the current plateau* (placed somewhere between -0.2 V and -0.8 V; depending upon the amount of O_2 present).
- *Set the electrode voltage to the value identified in the current plateau*.
- *Gently pull the electrode upwards to insert a root through the sleeving electrode*. Care must be taken when inserting the root as these can be squashed or bent by mistake, restricting internal longitudinal O_2 diffusion.
- *Once the electrode is positioned in the target region of the root, measure the diffusion current after the the system is at equilibrium* (c. after 30 min).
- *Move the electrode to a new position* and repeat the step above.
- *After measurements, move the electrode away from the root* (i.e., move it to the bottom of the acrylic chamber) and measure the residual current in the deoxygenated solution once the system is at equilibrium (c. after 30 min).
- Harvest the root used for ROL measurements and determine the root diameters at the different positions that were used for ROL determination by using a calliper.

General considerations

- Provide PAR illumination and leave the shoot of the plant intact to guarantee photosynthesis and O_2 transport.
- Measurements should be done at a set temperature since root growth, respiration and diffusion processes are all affected by temperature.
- In a sufficiently deoxygenated solution, the equilibrium current will probably be between 0.01-0.05 µA.
- It is important to check plateau potentials frequently, particularly when moving the electrode to other roots or when changing the O_2 regime around the shoot. The plateau shift towards lower or higher voltages with less or more O_2 in the solution, respectively.
- It is important to keep the acrylic chamber filled with deoxygenated solution to the top to prevent air mixing.

Relevant literature

- Armstrong W, Wright EJ. 1975. Radial oxygen loss from roots: the theoretical basis for the manipulation of flux data obtained by the cylindrical platinum electrode technique. *Physiologia Plantarum* 35: 21–26.

- Armstrong W. 1979. Aeration in higher plants. *Advances in Botanical Research* 7: 225-332.

Supplementary information S3: Clark type O_2 microsensors or Microoptodes

List of equipment needed

- Clark type O_2 microsensors or microoptodes (tip diameter 10, 25 or 50 µm)
- pA meter or optode meter
- Micromanipulator
- Stereo microscope on a boom-stand
- Transparent flume (1-2 L in volume)
- Grid (nylon or steel) to fix the sample using rubber bands
- Glass or acrylic blocks to elevate the grid above the bottom of the flume
- Peristaltic pump
- Blu-Tack putty
- PAR lights
- Air pump with air stone
- Calliper

List of chemicals needed

- High-purity N_2 gas
- Ascorbate and (Na or K)-hydroxide pellets for zero calibration

General procedure

- *Connect the O_2 microsensor to the pA meter and let it polarize for a minimum of 2 h* (connect it the day before, if possible). *If using an O_2 optode, no polarization time is required*.
- *Calibrate the sensor at air equilibrium* (DI water gently purged with air for 1 h) at the target temperature. Make sure that the temperature is exactly the same as for the planned experiment as the temperature coefficient of the O_2 sensor is *c.* 2-3 % $°C^{-1}$ (too vivid purging is going to cool the solution due to evaporation).
- *Calibrate the sensor at zero O_2* at the exact same temperature. Prepare a solution the day before using approximately 100 mL of DI water (fill a container to the very top to avoid gaseous headspace). Add 3 pellets of hydroxide and *c.* 2 g of ascorbate. Shake well and leave it overnight. The solution will remain at zero O_2 for some weeks if store dark (wrap Al foil around the container).
- *Select a suitable plant and identify the target root.* Cut off all other roots near the root-shoot junction to avoid that these roots oxygenize the medium. If needed, part of the shoot can be severed as well but make sure to leave back stumps of approximate 10 cm in length. Be careful not to bend the target root in this process, as this will block the aerenchyma and thereby impede longitudinal internal diffusion of O_2.
- *Carefully mount the root on the grid using pieces of rubber band woven into the grid*. The rubber bands should just be tightened sufficiently to prevent movements during

measurements. Like above, any obstruction of the aerenchyma will lead to a decrease in internal longitudinal O_2 diffusion. Remember to keep the root moist during this process!

- *Position the grid with the root attached inside the flume* onto two blocks of acrylic or glass using Blu-Tack putty inside the flume. The grid must be elevated above the bottom of the flume to enable flow both above and below the root.
- *Fill the flume with deoxygenated DI water* (below 3 µmol L^{-1}) using a siphon to minimize back-mixing of O_2. Fill it so that half of the root is cover with water; this prevents desiccation of the root.
- *Position the O_2 microsensor on the root surface using the micromanipulator.* If using a motorized stage, remember to set vertical position to zero. Positioning of the microsensor is greatly aided by a stereo microscope.
- *Fill the flume completely with deoxygenated DI water* (the root should be covered with a minimum of 10 mm of water).
- *Connect the flume to the reservoir of deoxygenated DI water and start the peristaltic pump so that the water is renewed c. once every 5-10 minutes.* In place of a peristaltic pump, gravity flow can be used instead.
- *Take measurements of O_2 (µmol L^{-1}*) along the root starting at the root surface and then moving outward into the DBL at discrete steps of 25 to 50 µm. 4-6 point within a distance of 250 µm from the root surface is sufficient.
- *Harvest the root and determine the root diameters at the different positions* that were used for ROL determination by using a digital calliper.
- *ROL is calculated using the approach of Henriksen et al. 1992.*

General considerations

- Measurements should be conducted at a set temperature since root growth, respiration and diffusion processes are all affected by temperature.
- If the external O_2 concentration in the flume exceeds 3-5 µmol L^{-1}, low rates of ROL cannot be accurately detected.
- In contrast, trace amounts of O_2 enables detection of O_2 consumption by the outer cell layers in situation where a tight root barrier to ROL has been formed; see example in Pedersen et al. 2021.

Relevant literature

- Colmer TD, Winkel A, Kotula L, Armstrong W, Revsbech NP, Pedersen O. 2020. Root O_2 consumption, CO_2 production and tissue concentration profiles in chickpea, as influenced by environmental hypoxia. New Phytologist 226: 373-384.

- Henriksen GH, Raman DR, Walker LP, Spanswick RM. 1992. Measurement of net fluxes of ammonium and nitrate at the surface of barley roots using ion-selective microelectrodes II.

Patterns of uptake along the root axis and evaluation of the microelectrode flux estimation technique. Plant Physiology 99: 734-747.

- Manzur ME, Grimoldi AA, Insausti P, Striker GG. 2015. Radial oxygen loss and physical barriers in relation to root tissue age in species with different types of aerenchyma. Functional Plant Biology 42: 9-17.

- Pedersen O, Nakayama Y, Yasue H, Kurokawa Y, Takahashi H, Floytrup AH, Omori F, Mano Y, Colmer TD, Nakazono M. 2021. Lateral roots, in addition to the main axis of adventitious roots, form a barrier to radial oxygen loss in *Zea nicaraguensis* and a chromosome segment introgression line in maize. New Phytologist 229: 94-105.

Supplementary information S4: Planar Optodes Method

List of equipment needed

For construction of the planar optodes

- Glass plates (200x300 mm)
- Coating device (or a knife)
- Glass pipette

For measurements

- Chamber with a transparent glass front
- Blue LED light source (450 nm)
- LED excitation short pass filter (475 nm)
- High-end digital camera with macro lens
- Emission long pass filter (530 nm)
- LED triggerbox
- Look@RGB software
- PAR lights (optional)

List of chemicals needed

- Mylar foil (125-175 µm thickness)
- Polystyrene
- PtTFPP (indicator)
- Macrolex Yellow (antenna dye)
- Dichloromethane
- Silicone ELASTOSIL N189 black
- Hexane

General procedure

Planar optodes construction

- *Prepare 10% polystyrene matrix in dichloromethane.* Stir the solution overnight. It can be stored at 5 °C for several months.
- *Prepare a fresh small amount of sensing cocktail by dissolving 1 g L^{-1} PtTFPP and 1 g L^{-1} Macrolex Yellow in the polystyrene matrix.* 20 mL of sensing cocktail corresponds to about 6-8 optodes (80x 150 mm). Stir the solution for 1-2 h.
- *Clean the mylar foil with soap and water and leave it to dry.* Be careful not to scratch the foil as it ruins the quality of the optode.

- *Position the mylar foil on the glass plate using acetone (or water).* The strong capillary forces snap the foil to the glass plate preventing that air bubbles are trapped between the foil and the glass plate.
- *Pour 1-2 mL of the sensing cocktail on the top of the foil using a glass pipette and coat the foil using the coating device. Let it dry overnight.* Be quick during the coating, dichloromethane is highly volatile. Apply sufficient amount of the cocktail (depending on the size of the optode) to guarantee a coat of 100-150 µm thick, which will result in a final thickness of 10-20 µm once the liquid has evaporated.
- *Prepare the silicone coating solution by dissolving the black silicone in hexane in a ratio of 1:5.* The dissolution can take ca. 1-2 h.
- *Coat the planar optode using the coating device. Let it dry overnight.* Use a thickness of 150-200 µm for a final thickness of the coating of 40-60 µm.

Measurements

- *Fix the planar optode on the glass front of the chamber using water.* Make sure to avoid air bubbles between the sensor foil and the glass.
- *Calibrate the planar optode.* Use a gas mixer to prepare solutions of known O_2 concentrations. For the zero O_2 solution, anoxic soil or medium can be used. The planar optode can be calibrated based on 3-point calibration (e.g. 0, 50 and 100% of air equilibrium).
- *Position the plant and fill the chamber with anoxic soil or medium.* In case of anoxic solution, make sure to slightly and homogenously position the roots toward the planar optode. Remember to measure root diameter using a calliper or taking a photo of the plant before filling the chamber.
- *Leave the system to equilibrate*. The time required depends mainly on the O_2 consumption rate of the soil/medium.
- *For calibration and actual measurements, take images in darkness.* Use manual setting for the camera and the Look@RGB software to adjust LED intensity and camera aperture (e.g. 300 mA, f2.8, 1/6''Tv). Images collected are automatically divided into RGB channels by the software.

Image analysis

- *Use ImageJ for image processing.* Based on the ratiometric approach, the intensity ratio between the Red and Green emission images is used to determine the O_2 concentration, following the equation:

$$\mathrm{R} = \frac{\mathrm{Red} - \mathrm{Green}}{\mathrm{Green}}$$

Where R is the pixel intensity ratio and Red and Green the intensities of the red and green channels, respectively.

- *Oxygen planar optodes are calibrated using a modified Stern-Volmer equation.* The constants K_{sv} and α can easily be obtained based on the 3-point calibration as follows:

$$K_{sv} = \frac{R0(C2 - C1) - (R1C2 - R2C1)}{(R1 - R2)C1C2}$$

$$\alpha = \frac{R1(1 + K_{sv}C1) - R0}{R0K_{sv}C1}$$

Where R0, R1 and R2 correspond to the intensity ratio of the 3-point calibration (R0= zero O_2 soil/medium) and C1 and C2 refer to the O_2 concentration used for R1 and R2.

- *Oxygen concentration (C) is obtained based on the intensity ratio of the sample (R), R0 and the constants K_{sv} and α*, and it is calculated as follows:

$$C = \frac{R0 - R}{K_{sv}(R - R0\alpha)}$$

- *ROL can be calculated based on the root radius and the O_2 consumption rate in the soil/medium*. The following equation is applied:

$$ROL = \Phi d\delta\left(1 + \frac{\delta}{2r}\right)$$

Where Φ is the soil porosity, d is the O_2 consumption rate, δ the distance between the root surface and the extinction of O_2 in the soil/medium (obtained from profiles on planar optode images) and r is the root radius. d can be calculated based on the O_2 penetration depth in the soil/medium using the following equations:

$$d = \frac{DOU}{O_2 \text{ penetration depth}}$$

$$DOU = \Phi D\frac{\Delta C}{\Delta z}$$

Where DOU is the diffusive O_2 uptake, D is the O_2 diffusion coefficient and ΔC is the concentration gradient within the Δz 2-point distance in the diffusive boundary layer.

General considerations

- Prepare the planar optode under the fume hood and in a dust-free environment.

- Other indicators are commercially available. PtTFPP offers longer phosphorescent lifetimes and high resolution at low O_2. Therefore, it is ideal for monitoring O_2 dynamics at the root-rhizosphere interface.
- Instead of the coating device, a knife can be used for the optode preparation and coating.
- The coating must be made with a constant velocity. Some practice is needed to make homogeneous optodes.
- Clean the coating tool as soon as possible. The indicator and the dye are hard to remove from the surfaces. Use acetone or a strong solvent for cleaning.
- Planar optodes can be stored at room temperature and in darkness for months.
- Provide PAR illumination to plants for long-time measurements.
- The ratiometric approach is relatively insensitive to non-homogeneous optodes (e.g. uneven luminophore distribution).
- Conduct calibration and measurements at the same temperature. The reaction chemistry responds to temperature.
- Medium/soil should be anoxic, free from toxins that can potentially affect the plant and consume O_2. This is an important requirement for measuring ROL.
- More images can be taken for each replicate. An average can be obtained during the image processing to reduce image noise.
- Additional noise can be adjusted by pixel binning. It reduces the spatial resolution of the final image but a 2x2 binning is a good compromise.

Relevant literature

- Santner J, Larsen M, Kreuzeder A, Glud RN. 2015. Two decades of chemical imaging of solutes in sediments and soils–a review. Analytica Chimica Acta 878: 9-42.

- Larsen M, Santner J, Oburger E, Wenzel WW, Glud RN. 2015. O_2 dynamics in the rhizosphere of young rice plants (*Oryza sativa* L.) as studied by planar optodes. Plant and Soil 390(1): 279-292.

- Larsen M, Borisov SM, Grunwald B, Klimant I, Glud RN. 2011. A simple and inexpensive high resolution color ratiometric planar optode imaging approach: application to oxygen and pH sensing. Limnology and Oceanography: Methods 9(9): 348-360.
